# Dengue-Associated Secondary Hemophagocytic Lymphohistiocytosis Presenting as Persistent Cytopenia: A Case Report

**DOI:** 10.7759/cureus.110976

**Published:** 2026-06-16

**Authors:** Satya Sai Prapurn Raavi, Bhargavi M V, Viswanathan Pandurangan, Ramya Anandh, Rajkumar Mani

**Affiliations:** 1 Internal Medicine, Sri Ramachandra Institute of Higher Education and Research, Chennai, IND; 2 General Medicine, Sri Ramachandra Institute of Higher Education and Research, Chennai, IND

**Keywords:** dengue fever, hemophagocytic lymphohistiocytosis, hyperferritinemia, pancytopenia, secondary hlh

## Abstract

Hemophagocytic lymphohistiocytosis (HLH) is a rare but life-threatening hyperinflammatory syndrome caused by uncontrolled immune activation and increased release of pro-inflammatory cytokines, resulting in multiorgan dysfunction. Secondary HLH is often activated by infections, especially viral infections. Dengue fever, the most common arboviral infection in tropical regions, is an uncommon but known precipitating factor of HLH and continues to challenge diagnosis due to overlapping clinical and laboratory manifestations. We describe the case of a 23-year-old previously healthy woman who presented with high-grade fever, abdominal pain, and progressive cytopenias. Laboratory investigations demonstrated pancytopenia and elevated hepatic transaminases. Dengue IgM serology was positive, and other infectious etiologies were ruled out. Despite supportive management, the patient had persistent fever and deteriorating hematological parameters. Additional blood workup showed elevated serum ferritin, hypertriglyceridemia, hypofibrinogenemia, and increased D-dimer levels. Bone marrow examination showed hemophagocytosis, meeting five out of eight diagnostic criteria for HLH, hence confirming the diagnosis of dengue-associated secondary HLH. She was successfully treated with intravenous dexamethasone and gradual oral tapering that led to clinical improvement and recovery of hematological parameters. Early identification and rapid initiation of immunosuppressive therapy were key to preventing further clinical decline. This case underscores the need for a high index of clinical suspicion for HLH in patients with dengue infection presenting with febrile illness, progressive pancytopenia, and elevated ferritin levels. Differentiating severe dengue from dengue-associated HLH early is crucial because their treatment regimens vary. Early diagnosis and appropriate corticosteroid therapy can improve patient prognosis.

## Introduction

Dengue fever is an endemic arboviral infection in tropical and subtropical regions. Clinical manifestations may vary from a minor febrile illness to serious dengue with plasma leakage, hemorrhagic manifestations, and shock [1]. Dengue, which causes fever and hematological aberrancies (e.g., leukopenia and thrombocytopenia), infects thousands of people annually. However, dengue-associated hemophagocytic lymphohistiocytosis (HLH) is a rare presentation and is often underdiagnosed due to the overlapping clinical features [2].

HLH is a hyperinflammatory syndrome associated with excessive immune activation, aberrant macrophage proliferation, and elevated effector-level pro-inflammatory cytokine production and is a life-threatening disorder. This loss of cytotoxic function, due to aberrant function of natural killer cells and cytotoxic T lymphocytes, leads to chronic antigenic stimulation and hemophagocytosis [3]. HLH is classified into primary (familial), with gene defects affecting perforin-mediated cytotoxic pathways, and secondary (acquired), which is secondary to infections, malignancies, or autoimmune diseases [4]. Dengue-associated HLH is often underrecognized due to its clinical overlap with severe dengue, and its exact incidence remains uncertain. 

Viral infection is one of the most widespread trigger factors of secondary HLH [5]. As those classical associations have not changed, we must still consider Epstein-Barr as the main virus associated with the disease, despite more recent associations with other viruses such as cytomegalovirus, influenza, and arboviruses [6].

The immunopathogenesis of dengue-associated HLH is the sustained stimulation of viral antigen, with excessive release of cytokines, or cytokine storm [7]. High concentrations of interferon-γ, tumor necrosis factor-α, and interleukin-6 lead to macrophage activation and hemophagocytosis [8]. In the case of patients with dengue, clinical suspicion should be raised when they have persistent fever, progressive cytopenias, hyperferritinemia, and hypofibrinogenemia well into the course of the disease [9].

## Case presentation

A 23-year-old female with no comorbidities presented to us with diffuse abdominal pain for five days associated with nausea, vomiting, and some loose stools. Three days before admission, she developed high-grade fever with chills and rigors. No history of bleeding manifestations, rash, joint pain, altered sensorium, or recent drug exposure.

On presentation, vital signs were significant for a temperature of 101.4°F, pulse rate of 106/min, respiratory rate of 16/min, blood pressure of 100/60 mmHg, and oxygen saturation of 99% on room air. Her skin looked pale, but she was alert and oriented. Examination of the cardiovascular and respiratory systems was unremarkable. The abdomen was diffusely tender without guarding or rigidity. No hepatosplenomegaly was noted. Neurological examination was unremarkable.

On admission, the total blood investigations showed pancytopenia (Table 1), with hemoglobin 10 g/dL, total leukocyte count 3,620 cells/mm^3^, and platelet count 70,000/mm^3^. Peripheral smear revealed normocytic normochromic anemia with leukopenia and thrombocytopenia. The liver function test showed an elevated serum glutamic oxalacetic transaminase of 833 IU/L, with a normal bilirubin level. The result of the renal function test was normal.

**Table 1:** Serial laboratory parameters ALT, alanine aminotransferase; AST, aspartate aminotransferase; ESR, erythrocyte sedimentation rate; GGT, gamma-glutamyl transferase; HDL, high-density lipoprotein; IgG, immunoglobulin G; IgM, immunoglobulin M; INR, international normalized ratio; LDL, low-density lipoprotein; NS1, non-structural protein 1; PT, prothrombin time.

Category	Laboratory parameter	Patient's value	Reference range
Complete blood count	Hemoglobin	10.0 gm/dL	12.0-15.0 gm/dL
	White blood cell count	3620 cells/mm^3^	4000-11000 cells/mm^3^
	Platelet count (least)	0.70 lakhs/mm^3^	1.5-4.5 lakhs/mm^3^
	Platelet count (subsequent value)	1.43 lakhs/mm^3^	1.5-4.5 lakhs/mm^3^
Liver function tests	AST (initial value)	833 IU/L	<32 IU/L
	AST (subsequent value)	233 IU/L	<32 IU/L
	ALT (initial value)	131 IU/L	<33 IU/L
	ALT (subsequent value)	76 IU/L	<33 IU/L
	GGT	148 U/L	<40 IU/L
	Total bilirubin	0.67 mg/dL	<1.2 mg/dL
	Direct bilirubin	0.45 mg/dL	<0.30 mg/dL
	Total protein	6.3 g/dL	6.6-8.7 g/dL
	Albumin	2.9 g/dL	3.97-4.94 g/dL
Inflammatory markers	Ferritin	4038 ng/mL (1 in 50 dilutions)	13-150 ng/mL
	Ferritin (subsequent value)	1860 ng/mL	13-150 ng/mL
	Ferritin (pre-discharge value)	653.30 ng/mL	13-150 ng/mL
	ESR	82 mm/h	4-12 mm/h
	Procalcitonin	0.326 ng/mL	<0.046 ng/mL
Coagulation profile	INR	1.32	2-4
	PT	15.2 seconds	11.48-13.72 seconds
	Fibrinogen	120.8 mg/dL	250-520 mg/dL
	D-dimer	3.55 mg/L FEU	<0.55 mg/L FEU
Fasting lipid profile	Total cholesterol	88 mg/dL	<200 mg/dL
	Triglycerides	222 mg/dL	<150 mg/dL
	HDL cholesterol	8 mg/dL	>65 mg/dL
	LDL cholesterol	14 mg/dL	<100 mg/dL
Infections workup	Dengue NS1	Negative	<9 IgM units
	Dengue IgM	Positive (15.30 IgM units)	Negative <9, equivocal: 9-11, positive : >11
	Dengue IgG	Negative	Negative
	Scrub IgM	Negative	Negative
	Malaria antigen	Negative	Negative
	Leptospira IgM	Negative	Negative
	Blood culture	No growth	No growth
	Urine culture	No growth	No growth

Serologically, dengue IgM was positive (15.3), but dengue IgG and NS1 antigen were negative. Malaria, leptospira, scrub typhus test, and blood cultures were negative. The patient was treated conservatively with IV fluids, antipyretics, and regular monitoring of hematological parameters.

She developed intermittent fevers despite supportive management, and her cytopenias worsened. The repeated blood and urine cultures grew no organisms. On account of her prolonged fever with severe pancytopenia, secondary HLH was suspected. Additional laboratory analysis identified hyperferritinemia (4038 ng/mL), hypertriglyceridemia (222 mg/dL), and hypofibrinogenemia (120.8 mg/dL) (Table 1), meeting several HLH diagnostic criteria [10], with an H score of 230 indicating a 96%-98% probability of hemophagocytic syndrome [11]. 

**Figure 1:**
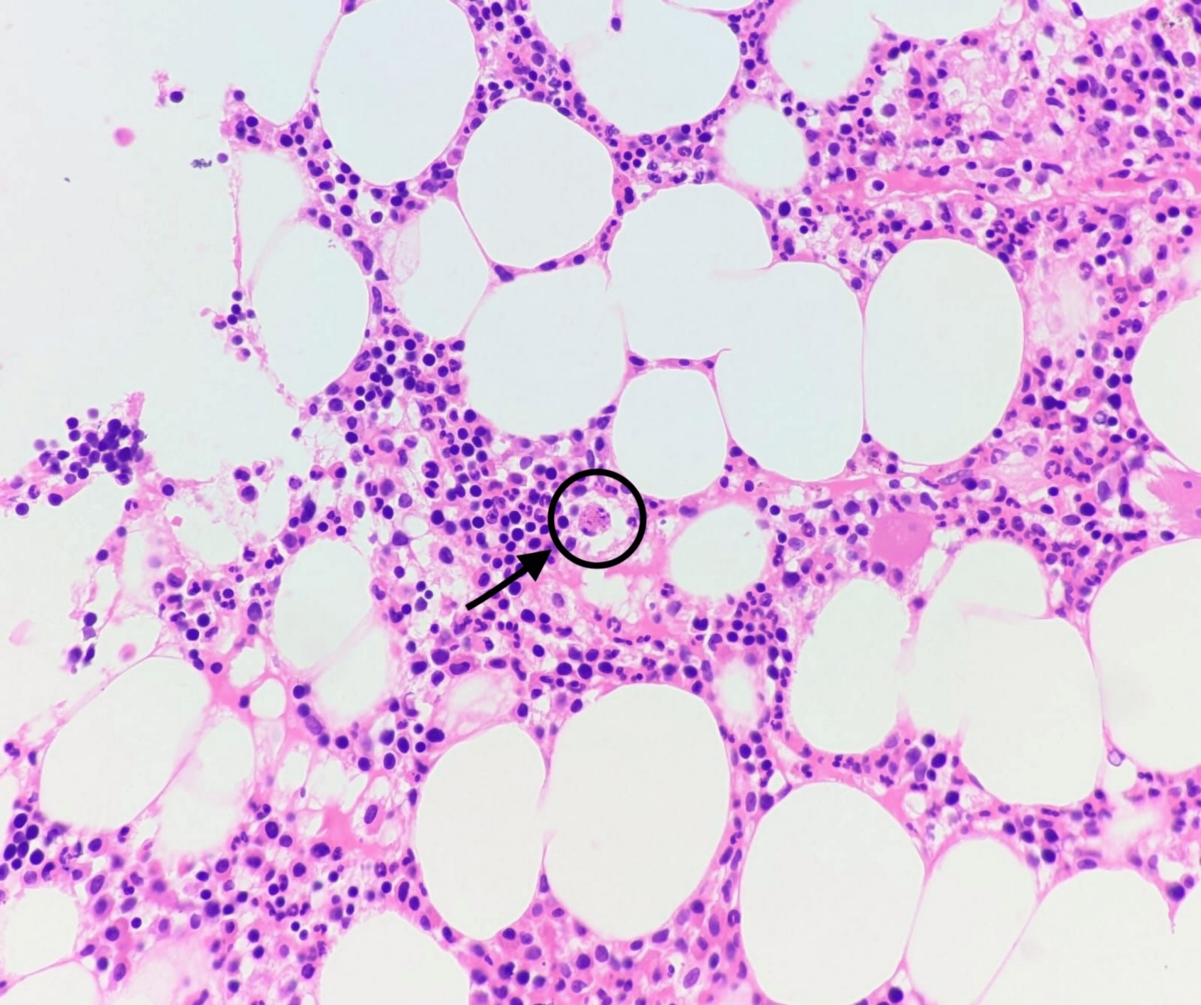
A bone marrow section stained with hematoxylin and eosin (40×) showing scattered histiocytes that exhibit hemophagocytosis (as highlighted) among the surrounding hematopoietic cells.

**Figure 2:**
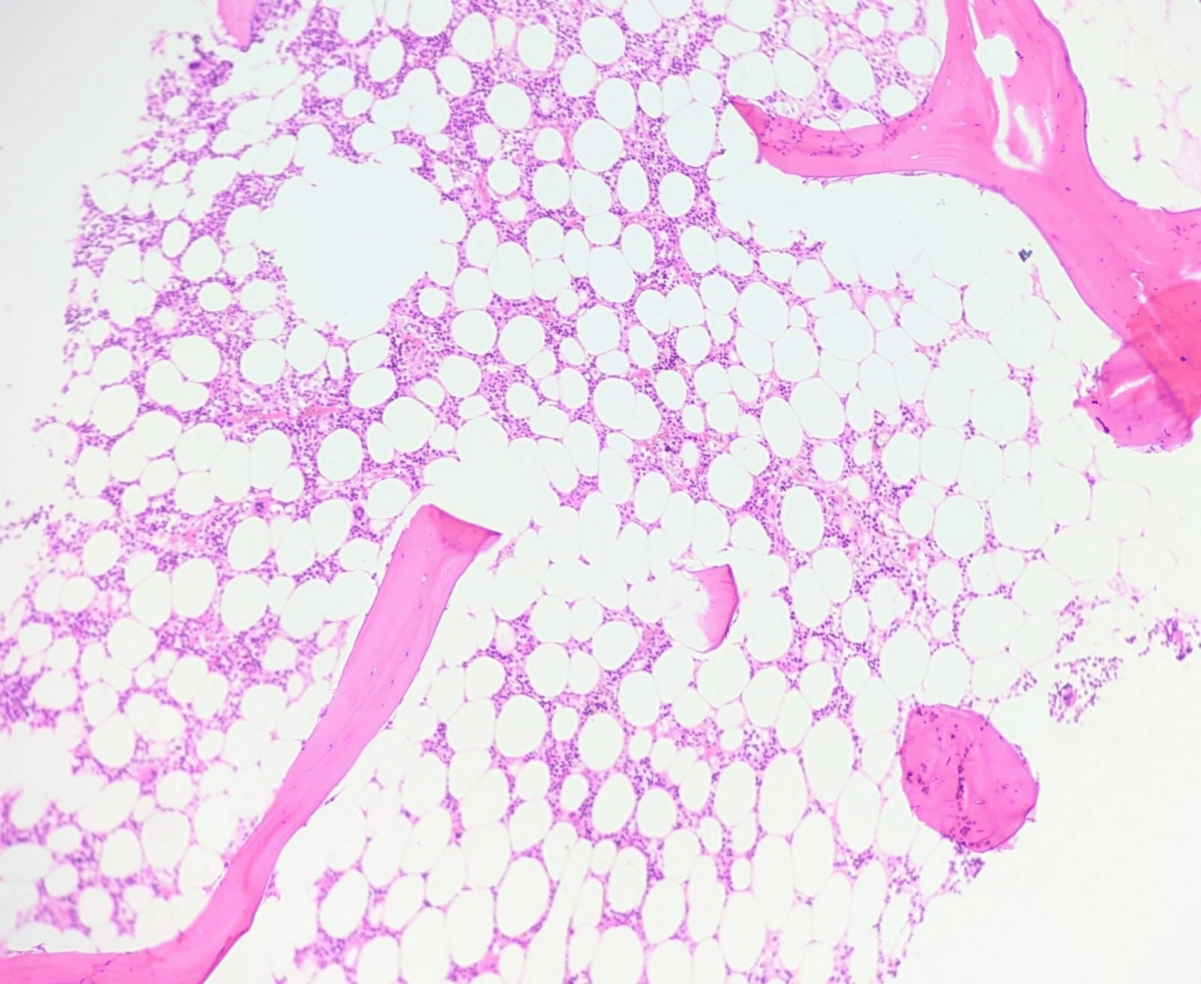
A hematoxylin and eosin-stained bone marrow section (10×) shows linear bony trabecular spaces surrounding variably cellular marrow spaces.

**Figure 3:**
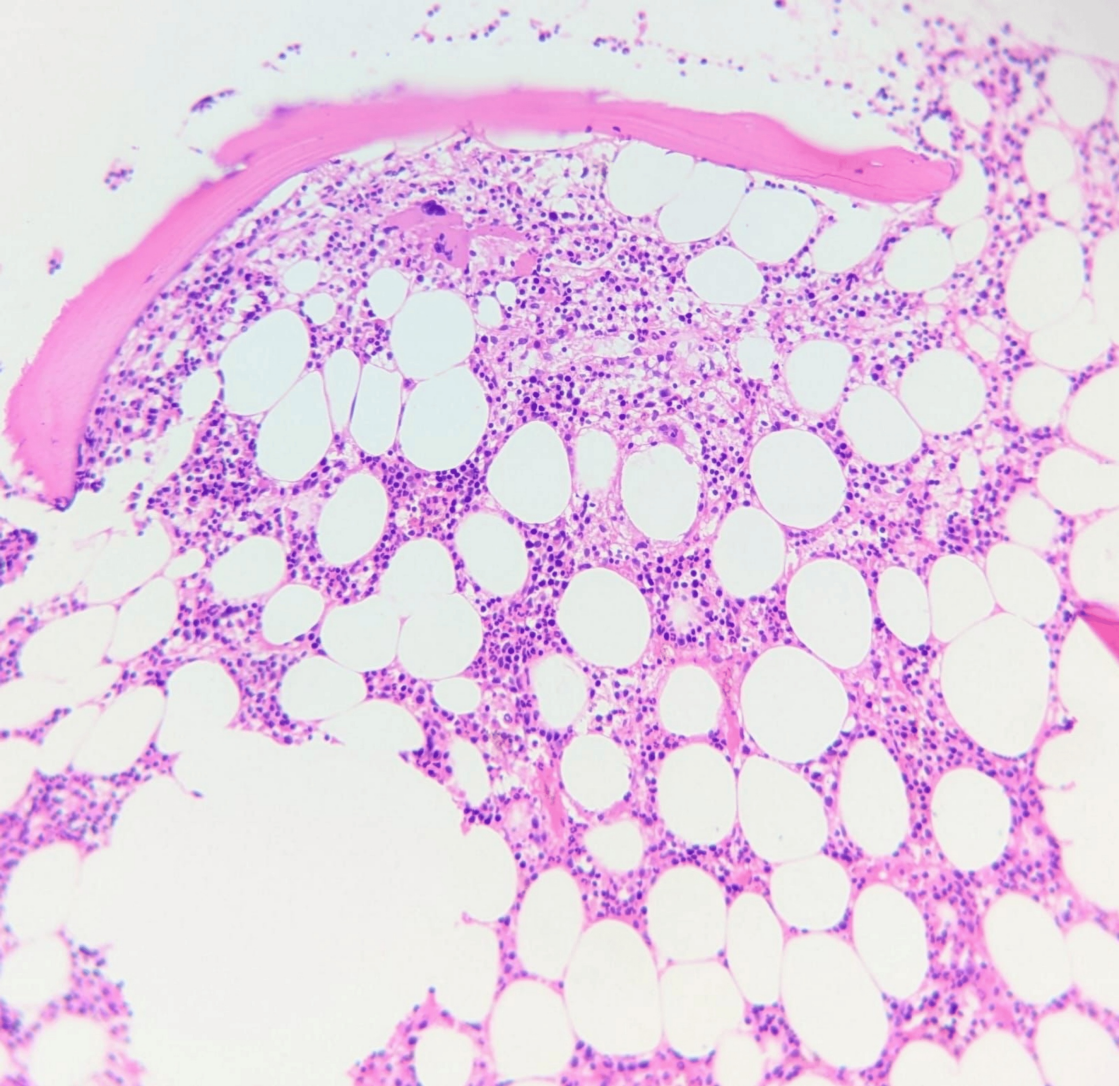
Hemotoxylin and eosin stain (20x magnification): Variably cellular marrow demonstrating normoblastic erythroid maturation, all stages of myeloid, and sufficient and normal megakaryocytes.

A bone marrow aspiration and biopsy were performed, which showed hypocellular marrow with hemophagocytes, confirming the diagnosis of secondary HLH (Figures 1-3). She was transferred to the ICU and initiated on IV dexamethasone (10 mg IV daily × 4 days, 6 mg IV daily × 2 days, followed by oral taper). Following initiation of therapy, she showed rapid clinical improvement with defervescence and recovery of hematological parameters. As a result, etoposide was not administered, and treatment was individualized based on the favorable response and underlying dengue-associated secondary HLH [12]. 

She was stable upon discharge and symptom-free upon follow-up.

## Discussion

Secondary HLH is a hyperferritinemic syndrome that occurs secondary to infections, malignancies, or autoimmune processes [4]. Viral infections are the most frequently encountered infectious triggers [5]. Although Epstein-Barr virus remains the most common causative agent, dengue virus is now recognized as an important cause of this phenomenon, particularly in endemic regions [2].

Dysfunctional cytotoxicity results in chronic immune activation among a pan-cytokine burst [7]. Malavige et al. reported immune dysregulation with increased proinflammatory cytokines in severe dengue infection [7]. Similarly, Martina et al. expanded on this by demonstrating an association between cytokine storm and the pathogenesis of dengue [8]. This over-stimulated immune response can then cause HLH in individuals who are sensitive to this phenomenon.

Persistent fever with worsening cytopenias, which are not typically observed in the classic febrile phase of dengue [9], should raise suspicion for HLH. Henter et al. described key diagnostic features of HLH, including hyperferritinemia, hypertriglyceridemia, hypofibrinogenemia, and hemophagocytosis [10]. Our patient fulfilled five out of the eight criteria for HLH, including markedly elevated ferritin. Allen et al. further reported that ferritin levels above 1000 ng/mL are strongly associated with HLH and may serve as a useful screening marker [13].

La Rosée et al. suggested therapeutic interventions for adult HLH, highlighting corticosteroids as the first-line agent for cases of intermediate severity [14]. Etoposide-based regimens may need to be considered in severe or refractory courses [14,15]. Concrete evidence of early immunosuppression reducing mortality was similarly shown by Schram and Berliner in their study [15]. The case highlights that dengue is a known risk factor for HLH and that patients presenting with hyperferritinemia and persistent fever should be closely evaluated for the presence of secondary HLH, especially in endemic settings.

## Conclusions

Dengue-associated secondary HLH is a rare and fatal complication. If a patient develops persistent fever, progressive pancytopenia, and hyperferritinemia with hypofibrinogenemia compatible with HLH, dengue infection should be suspected. Timely evaluation of the bone marrow and early treatment with steroids can significantly decrease mortality. Clinician awareness is crucial for early recognition and management.
